# Evaluation of SOVAT: An OLAP-GIS decision support system for community health assessment data analysis

**DOI:** 10.1186/1472-6947-8-22

**Published:** 2008-06-09

**Authors:** Matthew Scotch, Bambang Parmanto, Valerie Monaco

**Affiliations:** 1 Department of Biomedical Informatics, University of Pittsburgh, Pittsburgh, PA, USA; 2 Department of Health Information Management, University of Pittsburgh, Pittsburgh, PA, USA

## Abstract

**Background:**

Data analysis in community health assessment (CHA) involves the collection, integration, and analysis of large numerical and spatial data sets in order to identify health priorities. Geographic Information Systems (GIS) enable for management and analysis using spatial data, but have limitations in performing analysis of numerical data because of its traditional database architecture.

On-Line Analytical Processing (OLAP) is a multidimensional datawarehouse designed to facilitate querying of large numerical data. Coupling the spatial capabilities of GIS with the numerical analysis of OLAP, might enhance CHA data analysis. OLAP-GIS systems have been developed by university researchers and corporations, yet their potential for CHA data analysis is not well understood. To evaluate the potential of an OLAP-GIS decision support system for CHA problem solving, we compared OLAP-GIS to the standard information technology (IT) currently used by many public health professionals.

**Methods:**

SOVAT, an OLAP-GIS decision support system developed at the University of Pittsburgh, was compared against current IT for data analysis for CHA. For this study, current IT was considered the combined use of SPSS and GIS ("SPSS-GIS"). Graduate students, researchers, and faculty in the health sciences at the University of Pittsburgh were recruited. Each round consisted of: an instructional video of the system being evaluated, two practice tasks, five assessment tasks, and one post-study questionnaire. Objective and subjective measurement included: task completion time, success in answering the tasks, and system satisfaction.

**Results:**

Thirteen individuals participated. Inferential statistics were analyzed using linear mixed model analysis. SOVAT was statistically significant (α = .01) from SPSS-GIS for satisfaction and time (p < .002). Descriptive results indicated that participants had greater success in answering the tasks when using SOVAT as compared to SPSS-GIS.

**Conclusion:**

Using SOVAT, tasks were completed more efficiently, with a higher rate of success, and with greater satisfaction, than the combined use of SPSS and GIS. The results from this study indicate a potential for OLAP-GIS decision support systems as a valuable tool for CHA data analysis.

## Background

Data analysis in community health assessment (CHA) involves the collection, integration, and analysis of large numerical and spatial data sets in order to identify health priorities in community (or communities) of interest. Numerical data might include: vital statistics (e.g., birth, and death), registry data (e.g., cancer), inpatient and outpatient hospitalization data, and population (census) data. Spatial data might consist of spatial boundary files (such as 'shape' files) that contain geographically-defined coordinates. Combining numerical and spatial data is important for answering community health questions such as: "How does region A compare to its surrounding regions in relation to the incidence of asthma?" or "What are the top five causes of cancer deaths in a region, and how do these compare to the top 5 cancer deaths for the country?"

Geographic Information Systems (GIS) are applications that enable for management and analysis using spatial data [1]. Publications on the use of GIS in public health [2-8] suggest that it is viewed by many professionals as a useful tool for decision making. However, the technology has limitations in performing analysis of numerical data because of its traditional database architecture.

On-Line Analytical Processing (OLAP) is a multidimensional datawarehouse environment that is designed to facilitate querying of large numerical data [9,10]. Data in an OLAP data warehouse can be stored as a multidimensional cube in which all the numerical values are pre-calculated. While this can cause high memory requirements, querying only requires OLAP functions to fetch the data without the necessity to perform complex joins between tables. The software has been around since the 1990's and was initially very popular for use in the corporate environment to support high level decision making. OLAP has begun to gain popularity in the healthcare field but is still widely unknown to most health science researchers.

Coupling the spatial capabilities of GIS with a powerful technology for numerical analysis of On-Line Analytical Processing (OLAP), might enhance community health assessment data analysis. Examples of Online Analytical Processing-Geospatial Information System (OLAP-GIS) decision support systems have already been used for analysis in environmental health, community health, motor vehicle safety, and healthcare quality [11-14].

### Combining Numerical and Spatial Data for Community Health Assessment

Modern-day CHA professionals in developed countries frequently analyze public health data in order to identify health priorities. The steps in the process might be the:

• Identification of the spatial location of a geographic community using GIS or a paper map;

• Identification of health factors within the community using numerical data such as death counts, disease incidence or prevalence rates;

• Identification of the spatial location of bordering communities of interest using GIS or a paper map;

• Identification of health factors within bordering communities using numerical data such as death counts, disease incidence, or prevalence rates;

• Comparison of factors within the community against factors of the bordering community using statistical methods for adjustment and calculations such as relative risk and odds ratios;

• Viewing of results using tables, graphs, or spatial visualization.

The first step (of identification of the location of a geographic community) is a spatial component. This step represents the act of merely locating the area or region of interest on a map. The second step, identifying the health factors within the community, is purely numerical. For example, the ranking of top 5 diseases per 100,000 for a particular age category aggregated at the community level is a numerical process. However, the next step, identifying the bordering communities of interest is purely spatial. Like the first step, this can be done by using a map. The identification of health factors in these counties is purely numerical as in step 2. Statistical measures and adjustments are performed in order to determine health priorities.

Many community health experts use Information Technology (IT) for this type of data analysis. We conducted a survey of CHA professionals and found that many of them use software such as databases, statistical packages, and even GIS [15]. The potential for OLAP-GIS in community health data analysis is not well understood. We thus decided to conduct an evaluation comparing OLAP-GIS to information technology (IT) that is commonly used, including GIS and traditional analytical/statistical tools. We hypothesized that using an OLAP-GIS system instead of the combined use of a SPSS and GIS would greatly facilitate CHA data analysis when considering efficiency, accuracy and user satisfaction.

### SOVAT

**Figure 1:**
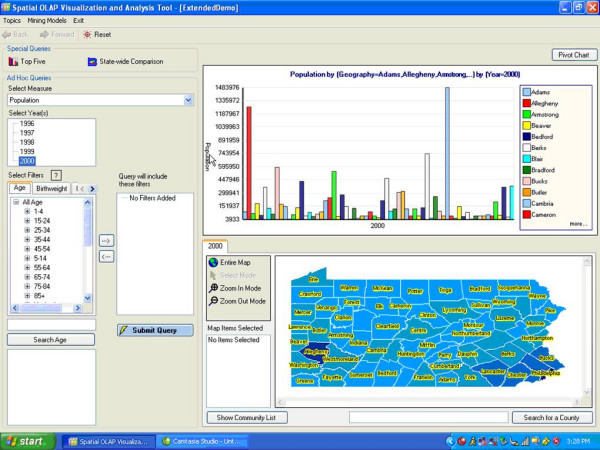
SOVAT interface.

At the University of Pittsburgh, we have developed an OLAP-GIS system called the Spatial OLAP Visualization and Analysis Tool (SOVAT) [16,17]. SOVAT is intended to support community health assessment data analysis. The system combines large amounts of health and population data and displays the information through a graphical user interface. The interface, developed using an iterative design approach [18], supports direct user manipulation as well as analysis of numerical and spatial components (Figure 1).

The SOVAT interface contains the ability to navigate through large public health data sets by using OLAP functions such as: *drill-down *(view more detailed data), *drill-up *(view more aggregated data), and *slice and dice *(view specific variables of data). In addition to these functions, SOVAT contains unique functions that are not standard in OLAP but were believed to enhance community health assessment. One such feature is called *drill-out*, which enables the user to click on a map object such as a county, and submit a query that contains both numerical and spatial aggregation. For example, to perform drill out on a 'region A', SOVAT would first identify the regions that border region A. This would be done through spatial analysis of the coordinates. Then the system would aggregate the numerical measures (such as an incidence rate) for each bordering region. This function enables the user to quickly perform comparisons of different geographical areas across different numerical public health measures.

We evaluated SOVAT against technology that we previously determined to be commonly used by CHA professionals, namely the combined use of SPSS statistical software and GIS software (referred to here as *SPSS-GIS*) [15], in order to understand its potential as a data analysis tool during community health assessments.

## Methods

Participants used both SOVAT and SPSS-GIS in a cross-over evaluation. Thirteen participants were enrolled in the study and included nine students and four faculty/researchers all within the health science schools at the University of Pittsburgh. The specific schools within the University's health sciences include the Schools of: Dental Medicine, Medicine, Nursing, Pharmacy, Public Health, and Health and Rehabilitation Sciences.

Participants were randomly assigned to the two study sequences: SOVAT → SPSS-GIS or SPSS-GIS → SOVAT. Depending on the sequence, they either used SOVAT or SPSS-GIS during period 1, given an interlude period between two to three weeks, and then used the other system during period 2.

### Recruitment and Setting

The participants were recruited via fliers that were posted around the University of Pittsburgh campus. The essential inclusion criterion was that the participants had experience using SPSS. Interested participants replying that they had never heard of SPSS or had used it a couple of times, were not enrolled in the study.

The study took place in a testing room within the Department of Biomedical Informatics at the University of Pittsburgh. The room contained a desk and chair for the participant, a laptop computer, and an overhead screen and projector.

### Software used in the Study

The current technology (SPSS-GIS) comprised two separate software applications: SPSS statistical software 13.0 and ArcView 9.1. SPSS, GIS, and SOVAT applications were all run locally off of the laptop during the evaluation.

### Study Procedures

Before entering the testing room, participants were asked to complete the informed consent form. Each session lasted approximately two and a half hours and was divided into two parts: training and evaluation. Once in the room, the participants were shown a pre-recorded instructional video that served as the introductory script for using the system being evaluated. They were allowed to take notes during this time. The content of the video, including the facets of the interface and the methodologies for producing queries, was deemed appropriate for use in the study by one of the co-investigators (VM) who is an expert in Human-Computer Interaction (HCI). After watching the video, the participants were given two practice tasks to solve using the system. After completing each task, they were shown a video solution for that task.

The participants were then given five problem solving tasks to answer using either SPSS-GIS or SOVAT (Table 1). Nielsen mentions that the tasks used during an evaluation study should be representative of real-world system use [19]. In order to ensure this, the tasks used in this study were deemed appropriate by an expert community health assessment researcher. They consisted of: performing local and state-wide comparison of geographic areas, ranking of diseases or geographic areas based on health measures, and defining and comparison of customized geographic communities. For the two systems, it was decided to make the task similar but not identical. So that the participants would not all receive the same ordering of tasks, Balanced Latin Squares (BLS) was used. Participants were randomly assigned to an ordered row of tasks. Camtasia screen capture software (TechSmith Corporation, Okemos, MI) was used to record their interaction while the external microphone captured their verbal thoughts.

**Table 1:** The five community health assessment tasks used in the evaluation study.

How does the outpatient rate per 1,000 of Warren County in 1998 compare to the outpatient rates per 1,000 in 1998 of the different counties that border it?
For this task the Eastern PA community is defined by the following counties: Bucks, Carbon, Lehigh, Monroe, Northampton.
The Northern PA community is defined by the following counties: Susquehanna, Bradford, Tioga, Potter, and McKean.
Compare the cancer incidence rate per 100,000 of female "Malignant Neoplasm of Colon" in 2000 between Eastern PA and Northern PA. Which counties not included in these communities border both of these two communities?

How does the cancer incidence rate per 100,000 in 1999 of Males Aged 75–84 in Indiana County compare to the cancer incidence rates per 100,000 (in 1999 of Males Aged 75–84) of the different counties that border it?
For the county with the highest rate, how does this rate compare with the state-wide rate for cancer incidence per 100,000 in 1999 of Males Aged 75–84?

What are the top 5 counties of deaths per 100,000 of "respiratory system" diseases 2000? Does one part of the state appear to contain the top 5 counties?

How does the Inpatient LOS (Length of Stay) per 1,000 in 2000 for females compare between Elk and Clarion Counties? For the county with the higher rate, what are its top 5 municipalities with Inpatient LOS per 1,000 in 2000 for females? Do all these municipalities border one another?

Once the participants completed the five tasks, they were asked to complete the IBM Post-Study System Usability Questionnaire (PSSUQ) [20]. This is mainly a close-ended questionnaire that has been found to be both a reliable and valid instrument for measuring user system satisfaction [20]. The PSSUQ is oriented in a 7-point Likert Scale format with lower numbers indicating higher levels of satisfaction. In addition to measuring overall system satisfaction, the questionnaire can be divided across three categorical areas: system usefulness, information quality, and interface quality.

After completing the second session, participants also completed a short one-on-one interview that lasted less than five minutes. The purpose of this interview was to go beyond the numeric responses from the satisfaction questionnaire and obtain more qualitative feedback regarding their attitudes towards both systems.

### Objective Measurements

The researchers believed that two essential criteria for determining system potential were *efficiency *and *accuracy*. Efficiency is a well-defined usability metric [19] and is often represented in the literature as time to task completion. Accuracy is an especially important criterion in community health assessment. Both efficiency and accuracy likely lead to a greater sense of confidence (i.e. positive feeling when using the system) and ultimately greater system use. In addition, the allocation of both financial and human resources for community health improvement is often based on conclusions drawn from data analysis. Software applications that lead to erroneous results and conclusions, could lead an inappropriate use of resources (of both time and money). The variables are described as:

• Time to complete each task (Efficiency) – This measure was defined by the time between when a participant finished reading the question to when the participant indicated he/she was done. The use of screen capture software allows one to measure the participant's time for each task. This screen capture method is also non-intrusive.

• Answer to Problem (Accuracy) – An answer was defined as the action of the participant verbalizing an answer to all the questions in the task followed by saying that they were 'done'. The answer did not have to be the same as what was currently being shown on the screen at the time. The participant had to answer all parts of the question correctly to successfully answer the task.

### Subjective Measurements

As mentioned, the PSSUQ was used for subjective measurement analysis of user satisfaction. A brief post-study interview was also conducted immediately following the completion of the second session. The question posted to every participant was "Which software system did you like better and why"? User preference was identified from the responses.

### Statistical Analysis

Both descriptive and inferential statistics were calculated for analysis purposes. Descriptive statistics were used for time, answer, satisfaction, and user preference. Inferential statistics were calculated by conducting mixed model analysis. This method enabled for design, period, and intervention effects to be identified across the variables *time *and *user satisfaction*. Statistical analysis was conducted using SPSS 13.0 for Windows.

## Results

### Time

**Figure 2:**
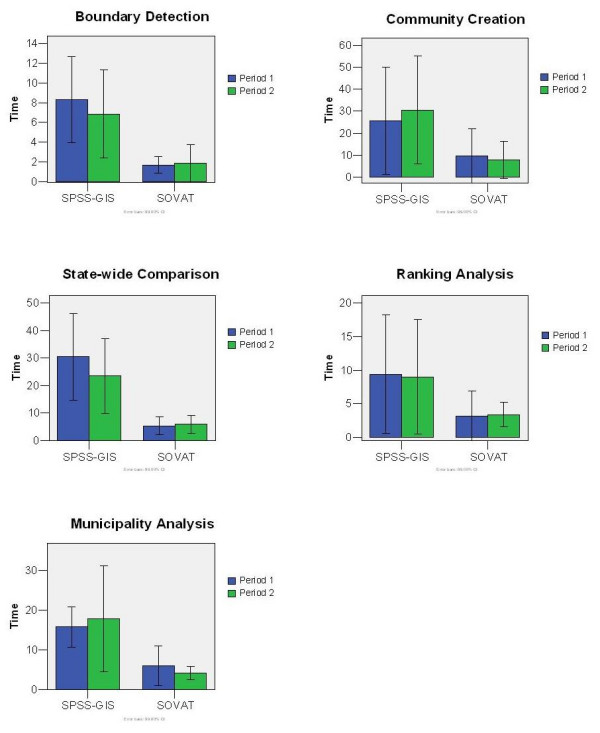
Mean time per task per period for SOVAT and SPSS-GIS.

Figure 2 shows the mean and 99% confidence interval for time rounded to the nearest minute. The results are shown by task by period. The five tasks are named based on their most distinguishable characteristic and are: boundary detection, community creation, state-wide comparison, ranking analysis, and municipality-level analysis.

### Success Rate

**Figure 3:**
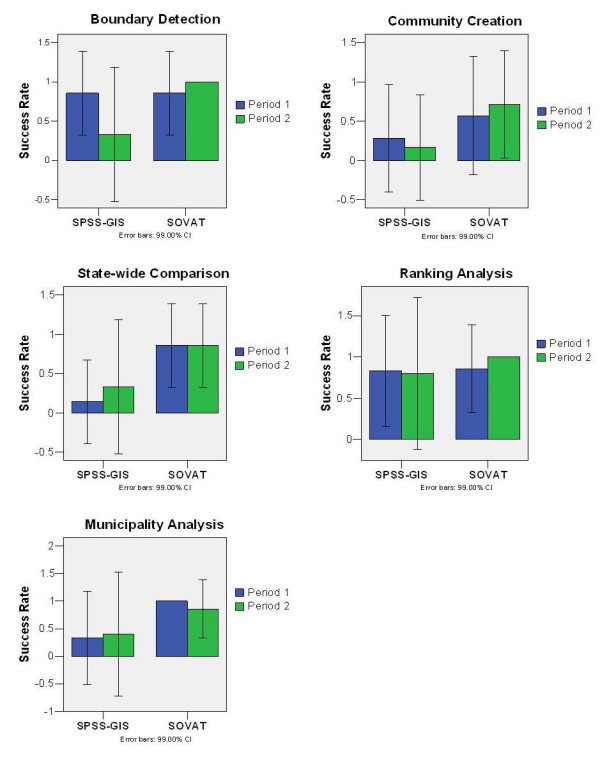
Success Rate per task per period for SOVAT and SPSS-GIS.

Figure 3 shows the success rates for the study. The success rate is equal to the number of tasks answered correctly divided by the number of tasks attempted. For SOVAT, all tasks were attempted. For SPSS-GIS, two participants attempted only three of the five tasks.

The bars indicate that the participants were more accurate using SOVAT than SPSS-GIS, yet the overlapping 99% confidence intervals suggests that the differences are not significant. Examining the specific tasks, the community creation task and the state-wide comparison were the most difficult tasks to perform using SPSS-GIS.

### User Satisfaction

**Figure 4:**
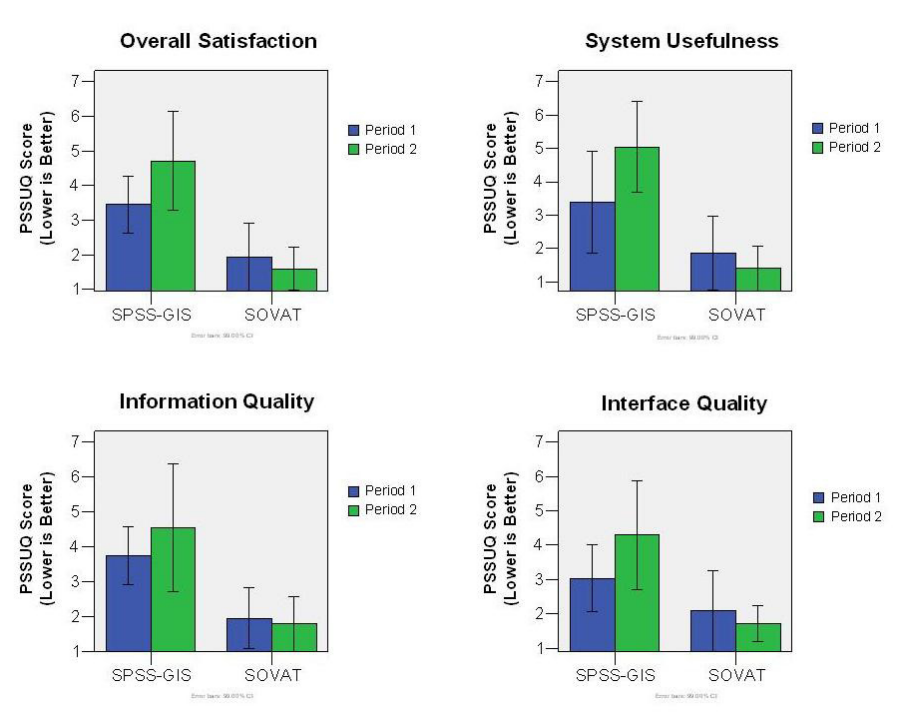
Satisfaction scores by period (A lower number is better).

Figure 4 shows the mean and 99% confidence intervals for the PSSUQ, with overall, as well as the satisfaction categories, by period. As mentioned, lower scores indicate higher levels of satisfaction.

The subjective data shows that SOVAT is perceived as more satisfactory across all periods and satisfaction categories than SPSS-GIS. Analyzing the three specific categories (not shown), system usefulness showed the greatest mean difference, while interface quality had the smallest mean difference.

### User Preference

Before completing the study, participants were asked additional questions such as, "What system did you like better and why?" In total, twelve of the thirteen participants (92%) preferred SOVAT, while one of the thirteen participants (8%) preferred the combination of SPSS and GIS.

The individual responses from the post-study interview were then categorized into groups. A participant could have more than one response if they commented on more than one aspect of the system. The counts for these groups and some example responses are shown in Table 2.

**Table 2:** Positive responses in relation to SOVAT during the post-study interview.

**Reason**	**Number of Participants Indicating**	**Participant Comments in Relation to this Reason**
Easier to Use	12	• "Streamlined for this purpose"
		• "Took less steps"
		• "Easier to go back"
		• "Very straightforward"
		• "Not as complicated"

Interface	6	Everything was together
		• "1 program vs. 2"
		• "Loved the interface; the layout; organized nicely; visually appealing"
		• "Layout was well designed"
		• "SOVAT interface looked better"

Information Access	4	• "Gave you the answer quickly"
		• "Easy to get information"
		• "Easier to find information"
		• "Finding data was easier"

Specific Features	6	• "Liked Search Boxes"
		• "Drill-out and community creation"
		• "Easy to create communities"
		• Drill-out helped for boundary detection"
		• "Rates already provided"

The majority of the positive responses towards SOVAT were in relation to its ease of use. The participants also liked the layout and design of the SOVAT interface better than SPSS-GIS. The most popular response in relation to this theme was that they like "1 program vs. 2." Hence, having to go back and forth between numerical and spatial data displays was not as popular as the combined interface of SOVAT.

Table 3 shows the negative responses towards SOVAT with the interface receiving the most feedback.

**Table 3:** Negative responses in relation to SOVAT during the post-study interview.

**Reason**	**Number of Participants Indicating**	**Participant Comments in Relation to this Reason**
Interface	4	• "Default setting. Allegheny was always darker"
		• "The bar chart was always changing color"
		• "No option to 'sort' bars in bar chart" "Map was not easy to navigate at Municipality level".

Information Access	1	• "Not as comprehensive as SPSS."
		• "Difficult to find information on screen"

### Mixed Model Analysis: Time

Mixed model analysis was used for obtaining inferential results for time and user satisfaction. The mixed model extends on the general linear model (GLM) to allow for fixed (treatment, period, group) and random effects (subjects) [21]. Both fixed and random variables are present in crossover designs and thus it was decided to use this model for inferential purposes. Table 4 shows the p-values for the three different effects in the study: *group *or sequence (SOVAT → SPSS-GIS, or SPSS-GIS→ SOVAT), *period *(period 1, period 2), and *intervention *(SOVAT, SPSS-GIS).

**Table 4:** Mixed model analysis of Time variable. Shown are p-values per effect per task.

	Boundary Detection	Community Creation	State-wide Comparison	Ranking Analysis	Municipality Analysis
**Group**	.338	.465	.250	.833	.269
**Period**	.429	.742	.244	.944	.953
**Intervention**	.000	.001	.000	.001	.000

At the .01 level, there is no group effect for any of the tasks. This indicates that the participants were sufficiently randomized to each group in relation to the variable *time*. The p-values for period indicate that there is no period effect for time. This indicates that the period (1 or 2) does not effect the time to complete the tasks. The intervention effect is significant at the .01 level. This indicates that the type of system used (SOVAT or SPSS-GIS) impacted the time to complete the tasks. As supported by the descriptive results, the participants completed the tasks in much shorter time with SOVAT than when they used a combination of SPSS and GIS.

### Mixed Model Analysis: User Satisfaction

The mixed model results for user satisfaction are shown in Table 5.

**Table 5:** Mixed model analysis of User Satisfaction.

	Overall Satisfaction	System Usefulness	Information Quality	Interface Quality
**Group**	.004	.003	.108	.008
**Period**	.080	.072	.283	.125
**Intervention**	.000	.000	.000	.000

The group effect corresponds to the treatment* period interaction which is an alias for a carryover effect [22]. As can be seen, the group effect is significant at the .01 level for overall satisfaction, system usefulness, and interface quality. It is not significant for information quality (p = .108). This indicates that a carryover effect was present in relation to participant responses on the satisfaction questionnaire.

In relation to period effect, there is no significant effect at the .01 level, suggesting that the period does not influence the satisfaction level of the participant. The intervention effect is significant at the .01 level for all satisfaction categories. This supports the mean results from the satisfaction questionnaire (Figure 4) that suggest that the participants are more satisfied with SOVAT than with SPSS-GIS.

## Discussion

The results seemed to favor SOVAT over the combination of SPSS and GIS. While the participants had all been familiar with SPSS, many had difficulty in using it for CHA data analysis that may require functions such as data aggregation and case selection, which are not often used during standard statistical analysis. For example, during the study it was common when using SPSS-GIS for the participants to:

• Open a GIS application and manually identify bordering areas on a map.

• Open SPSS and attempt to find a diagnosis among thousands of rows (or cases) of data.

• Type a complex "Select Cases" command that requires a statement to be syntactically accurate.

• Aggregate the selected cases by choosing appropriate break (or grouping) variables as well as the numerical measure on which to sum.

• Calculate the numerical rates.

• Return to the large SPSS file and specify a subset of the original selected cases.

• Aggregate the smaller subset of cases by selecting a different break variable and the numerical measures to sum.

• Calculate a new rate based on this latest aggregation.

Comparison between SOVAT and SPSS can be made by considering a specific task. For example, these steps are similar to solving the first task in Table 1 using SPSS (except for the second step which searches for a diagnosis). SOVAT on the other hand, requires fewer steps. For example, the first task listed in Table 1 compares one county to its bordering counties with respect to outpatient hospitalization rates. This can be completed in SOVAT by right-clicking on the county and using the *drill-out *function. SOVAT will use spatial boundary detection to identify the neighbors and then display the outpatient rates for comparison (screenshots of SOVAT solving a similar task can be seen in [16]).

The results did indicate a carryover effect from period 1 to period 2. There are many possible reasons for this. One may be the similarity of the tasks (they are similar, but not the same). That is, a participant might use SOVAT in period 1 and see similar tasks when using SPSS-GIS for period 2. The participant might believe during period 1 that they can easily complete these types of tasks but then have difficulty during the session using SPSS-GIS. As the charts in Figure 4 indicate, SOVAT was always better in period 2, while SPSS-GIS is always worse in period 2. This is consistent with the belief that SOVAT is perceived as more satisfactory than SPSS-GIS.

### OLAP-GIS Use in Community Health

A survey involving community health professionals in Canada showed that 70% of the respondents felt that GIS could enhance their community health decision making [23]. Despite its growing popularity, a significant limitation of GIS is that it is not designed to support numerical and multidimensional data exploration. Combining OLAP with GIS can enhance this process. Examples of using OLAP for public health decision making [24-29] show that members in the field are beginning to recognize it. Commercial products such as ESRI's OLAP add-on for ArcGIS and Microsft's OLAP add-on for MapPoint offer widespread availability and utilization of OLAP-GIS within the public health community.

## Limitations

The five community health tasks used in this evaluation were created by the researchers but then approved by a community health expert who was not part of the evaluation. The fact that the researchers created the questions could be viewed as a potential bias in the study since ones could have been chosen to favor the use of SOVAT over SPSS-GIS during evaluation. The authors believe that since the five tasks for community health assessment were deemed to have validity by an expert, that these types of tasks are representative of common CHA problems. Another potential limitation is the omission of professional representative users. It would have been preferred to use actual professionals in academic settings, health departments, or government agencies who use a combination of SPSS and GIS for CHA data analysis. This would have strengthened the generalizability of the results to include working community health professionals. Unfortunately, time away from work and difficulty in identifying local professionals who met the specific participation requirement, made this difficult. It was believed that obtaining potential future users of the system, such as graduate students, researchers, and some faculty within the health sciences programs at the University of Pittsburgh was the best alternative. It is believed that this population provided excellent results on which to compare the two independent variables. In addition, difference in technical knowledge between the participants might also have impacted the results. Likely some participants had extensive experiences with statistical and GIS applications, while others had limited experience. Efforts to eliminate this disparity included inclusion criteria of SPSS proficiency and pre-evaluation videos to train the participants on SOVAT, SPSS, and GIS. Finally, the fact that SOVAT was developed by the evaluators could be viewed as a potential bias.

## Conclusion

The results from this study show potential for OLAP-GIS decision support systems for community health assessment data analysis. Using SOVAT, tasks were completed more efficiently, with a higher rate of success, and with greater satisfaction, than the combined use of SPSS and GIS.

Beyond community health assessments, there are many others areas within public health that could benefit from OLAP-GIS decision support systems. Some of these include: environmental health, cancer, and real-time disease surveillance. Additional work will focus on the potential of OLAP-GIS in these different domains.

## Competing interests

Dr. Bambang Parmanto and Dr. Matthew Scotch were developers of the SOVAT system.

## Authors' contributions

MS conducted the evaluation and drafted the manuscript. BP critically reviewed the manuscript and advised on the evaluation. VM critically reviewed the manuscript and advised on the evaluation.

## Pre-publication history

The pre-publication history for this paper can be accessed here:


